# Editorial: Parent-Offspring Integration: Gut Health and Physiological Functions of Animals

**DOI:** 10.3389/fvets.2021.808074

**Published:** 2022-01-05

**Authors:** Xiangfeng Kong, Rajesh Jha

**Affiliations:** ^1^Institute of Subtropical Agriculture, Chinese Academy of Sciences, Changsha, China; ^2^Department of Human Nutrition, Food and Animal Sciences, College of Tropical Agriculture and Human Resources, University of Hawaii at Manoa, Honolulu, HI, United States

**Keywords:** gut microbiota, parent-offspring integration, nutritional intervention, growth and development, intestinal health

## Introduction

Despite the lack of a clear definition, the term “gut health” in animal nutrition or its etiology encompasses several physiological and functional features, including nutrient digestion and absorption, host metabolism and energy generation, a stable and appropriate microbiota/microbiome, defense mechanisms (i.e., barrier function and mucosal immune mechanisms), and the interactions between these components (1). Among other gut health variables, the gut microbiota has strong metabolic activities and plays several important roles in animals and poultry. It includes the regulation of nutrient utilization and physiological functions of the host, including the digestion and absorption of nutrients, fermentation of complex macronutrients, and nutrient and vitamin production, contributing to the construction of the intestinal epithelial barrier, the development and function of the host immune system, competing with pathogenic bacteria to prevent their harmful propagation, and physiological metabolism in distal organs or tissues (2–4). In addition to the composition of the gut microbiota, small molecule metabolites derived from gut microbiota can enter into the systemic circulation and play regulatory roles signaling molecules or toxins, affecting the performance and health of animals (1). In this context, maintaining a healthy gut microbiota has become a prominent strategy to improve animal and poultry's health and production performance.

It is well-established that mammalian gut microbiota regulates host metabolism, and its composition varies in hosts in different physiological states, such as during the pregnancy progression. In addition, maternal physiological changes during pregnancy are highly related to the growth and development of the fetus, which might be influenced by the gut microbiota (5, 6). Indeed, maternal gut microbiota (e.g., *Firmicutes* and *Proteobacteria*) could colonize the fetal/neonatal gut during pregnancy (via the placenta) or lactation (via maternal milk and mother's feces) and affecting the offspring's growth and development and as well other physiological functions later in life (7). Also, it is well-known that the gut microbiota is involved in regulating various host gut functions, and the offspring's growth and development largely depend on maternal physiological changes during pregnancy and lactation (8). Studies have found that maternal microbiota in the gastrointestinal tract (GIT) could colonize in the fetus *in utero* and then could be transmitted to the offspring through direct contact during parturition or lactation (9, 10). Also, maternal nutrition and metabolic and physiological conditions are the pivotal factors in fetal development, production performance, and offspring's growth and development (11). Therefore, maternal nutritional intervention during pregnancy and the perinatal period has been used to enhance the maternal gut health and improve the gut health of their offspring later in life (12, 13). Similarly, early growth and development of the GIT of poultry have critical importance to nutrients utilize and optimize the nutrients. Unlike in animals, early nutrition programming using both *in ovo* and post-hatch feeding has been used in poultry to modulate the early growth and development of gut health and found to be an effective strategy (14). *In ovo* feeding with specific prebiotics and probiotics affects gut microbiota and metabolic profile, ileal-histomorphology, immune functions, and growth performance of poultry (15, 16). Therefore, it is crucial to understand the parent-offspring integration on animals' gut health and physiological functions and nutritional strategies for beneficial impacts in offsprings.

To highlight recent findings in the field, a Research Topic entitled “*Parent-offspring Integration: Gut Health and Physiological Function of Animals*” was organized and came up with 14 relevant articles covering different aspects of the topic for both animals and poultry.

## Nutritional Strategies for Parent-Offspring Integration on Gut Health and Physiological Function

During pregnancy, the maternal system can be influenced by several extrinsic factors; nutritional status is one of those that can program nutrient partitioning and ultimate growth, development, and function of the major fetal organ system (11). In addition, fetal growth and development are also associated with the fetal nutritional environment and could change as pregnancy progresses. Over the past few decades, different maternal nutritional strategies (i.e., energy intake and protein levels) have been gained interest to evaluate the fetal development and production performance of animals. For example, specific nutrients such as amino acids (methionine, cysteine, glutamine, and glutamate) could improve fetal development and thus influence the gut health of animals. These gastrointestinal proteins- or amino acids-fermenting bacteria have the potential function in the utilization and production of amino acids and microbial proteins, in turn, to feed the host in return (12). Similarly, dietary supplementation with probiotics (*Bacillus subtilis*) promoted growth performance, decreased diarrhea incidence, and ameliorated several indicators of intestinal health through the modulation of gut microbiota composition and metabolic activity in weaned piglets (Tian et al.). Interestingly, Guava leaf extract improved intestinal barrier function and enhanced the antioxidant ability of piglets challenged by enterotoxigenic *Escherichia coli* (Wang D. et al.). Also, *Macleaya cordata* extract combined with benzoic acid affected growth performance, immune response, and gut microbiota in weaned pigs (Wang F. et al.). These studies suggest that plant extracts have the potential to be used as gut health enhancers in pigs. As potential gut health enhancers, dietary feed supplements (including prebiotics, probiotics, synbiotics, and fatty acids) are gaining more attention to be used in maternal feeding programs in parent-offspring integration. These feed additives have been found to modulate microbial community, regulate the production of cytokines and antibodies, and improve gut development and the overall gut health of animals and poultry (17–19). Wang K. et al. evaluated the effects of dietary probiotics or synbiotics supplementation during gestation, lactation, and nursery periods on colonic microbiota, antioxidant capacity, and immune function of weaned piglets. This study found that dietary probiotics or synbiotics supplementation to sows (during pregnancy and lactation) and their offspring piglets could increase the beneficial bacteria such as *Bifidobacterium* and *Lactobacillus* and decrease the pathogenic bacteria *Escherichia coli* in the colon of piglets. In addition, dietary probiotics or synbiotics supplementation to sows and their offspring piglets could improve the immune response and antioxidant capacity of weaned piglets. The study also found that intestinal microbiota changes were correlated with alterations of immunoglobulins and cytokine concentration and antioxidant capacity of piglets. Similarly, Wang X.-L et al. evaluated *Lactobacillus delbrueckii* as a probiotic in weaned pigs and found that it can improve intestinal morphology and modulate the microbiota community to promote growth performance. To get a similar understanding of poultry, Dunislawska et al. used bioactive substances, such as prebiotics, probiotics, or synbiotics, to evaluate the molecular response in intestinal and immune tissues *in ovo* study. They found that prebiotics and synbiotics could improve the gut barrier integrity and lipid metabolism and upregulate the gut-immune-related genes in the large gut. Das et al. reviewed different aspects of *in ovo* feeding and its application for modulating the performance and gut health of poultry. Thanabalan and Kiarie reported that feeding polyunsaturated omega-3 fatty acids to broiler breeders modulates breeder GIT microbiota with consequences of microbial colonization and succession in chicks. Also, it impacts the indices of immunocompetence, skeletal, and GIT development in chick post-hatch. Earlier, Yang et al. characterized the intestinal microbial community in broiler breeders to better understand their population and functions. In another study, Hernandez et al. evaluated the effectiveness of whole yeast cells, peppermint oil, and γ-tocopherol in gestation and lactation on maternal oxidative stress and offspring growth from birth to market. This study found that dietary inclusion of whole yeast cells, peppermint oil, and γ-tocopherol in sow diets improved offspring performance during the suckling and post-weaning periods. Moreover, whole yeast cell and γ-tocopherol inclusion in sow diets during lactation showed heavier offspring, while prenatal and postnatal inclusion of peppermint oil had lightweight pig up to the market. Zhou et al. evaluated the effect of supplementing all-trans retinoic acid to Hoxa1^+/−^ pregnant sows and found that all-trans retinoic acid minimized the developmental defects of Hoxa1^+/−^ and improved the birth weight and ear defects of Hoxa1^−/−^ piglets. Qi et al. reported the characteristics of intestinal microbial succession and the correlation with the production of two important types of bacterial metabolites (short-chain fatty acids and bioamine) in piglets at the early growth stage. Zhu et al. studied the dynamic changes of metabolic profiles in maternal biofluids during the gestation period in a native breed (Huanjiang Mini-pigs) and found that there was a relationship with specific amino acids concentration in amniotic and allantoic fluid with the bodyweight of fetuses. On the other hand, Tang et al. reviewed the mechanisms of epidermal growth factor and their effects on animal intestinal phosphate absorption with the intention to highlight its role in gut health.

In conclusion, maternal nutritional programming could influence both parents' and their offspring's gut health and physiological functions. However, the underlying mechanisms of parent-offspring nutrient transportation still have remained unelucidated. Therefore, we should consider the parent-offspring integration, and more studies in this field are necessary to understand the exact mechanisms and functions and the long-term effects on gut health.

## Author Contributions

Both authors listed have made a substantial, direct, and intellectual contribution to the work and approved it for publication.

## Conflict of Interest

The authors declare that the research was conducted in the absence of any commercial or financial relationships that could be construed as a potential conflict of interest.

## Publisher's Note

All claims expressed in this article are solely those of the authors and do not necessarily represent those of their affiliated organizations, or those of the publisher, the editors and the reviewers. Any product that may be evaluated in this article, or claim that may be made by its manufacturer, is not guaranteed or endorsed by the publisher.
